# Assessment of antioxidant and antidiabetic properties of *Agaricus blazei* Murill extracts

**DOI:** 10.1002/fsn3.1310

**Published:** 2019-12-05

**Authors:** Qi Wei, Yishu Zhan, Bingzhi Chen, Baogui Xie, Ting Fang, Sadhana Ravishankar, Yuji Jiang

**Affiliations:** ^1^ College of Food Science Fujian Agriculture and Forestry University Fuzhou China; ^2^ Mycological Research Center Fujian Agriculture and Forestry University Fuzhou China; ^3^ School of Animal and Comparative Biomedical Sciences University of Arizona Tucson AZ USA

**Keywords:** *Agaricus blazei *Murill, antidiabetic activity, antioxidant activity, HepG2 cells, α‐glucosidase

## Abstract

*Agaricus blazei* Murill (ABM), a medicinal mushroom, has beneficial effects on various human metabolic diseases. The objective of this research was to evaluate the antioxidant and antidiabetic properties of ABM extracts (ethanol extract and ethyl acetate extract). The antioxidant activities of ABM ethanol extract (EE) and ethyl acetate extract (EA) were analyzed using 1,1‐diphenyl‐2‐picrylhydrazyl (DPPH), 2,2′‐azino‐bis (3‐ethylbenzothiazoline‐6‐sulfonic acid) (ABTS), and hydroxyl radical scavenging assays and the reducing power using K_3_Fe(CN)_6_ in vitro. Moreover, the effects of EE and EA on α‐glucosidase inhibitory activity and improving glucose uptake by HepG2 cells were investigated in vitro. The EA showed stronger antioxidant activity, as well as inhibition of α‐glucosidase, compared to EE. The analysis of glucose uptake by HepG2 cells showed that EA had significant glucose‐lowering activity and exhibited no difference compared to metformin. The results suggest that ABM extracts could improve the glucose uptake by HepG2 cells and thereby alleviate postprandial hyperglycemia. This investigation provides a strong rationale for further studies on the application of ABM to control type 2 diabetes.

## Graphical abstract

Agaricus blazei Murril extracts showed high antioxidant activity. Agaricus blazei Murril extracts demonstrated potential anti‐diabetic activity.

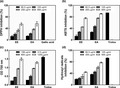

## 1 INTRODUCTION

Type 2 diabetes is characterized as insulin resistance, namely the body's sensitivity to reduced insulin levels, resulting in excessive accumulation of insulin in the plasma. Irregularly increasing glucose levels could lead to many chronic diseases (Gao, Guo, Qin, Shang, & Zhang, 2017; Wu, Shi, Wang, & Wang, 2016). Thus, the control of blood glucose level is a critical method to prevent the progression of type 2 diabetes and its complications (Martinez, Lockhart, Davies, Lindsay, & Dempster, 2018). Medicinal plants have been used for the treatment of type 2 diabetes. The active mechanism of medicinal plants seems to be directly on pancreas by stimulating it to produce insulin which increases its level in blood. Since the medicinal plants have less side effects, they can be potentially employed in developing new and better drugs for diabetes (Shukia, Sharma, Puri, Prabhu, & Murthy, 2000).

α‐glucosidase is an enzyme that plays a key role in the final step of digestive process, where this enzyme breaks down complex carbohydrates such as starch and glycogen into their monomers. Accordingly, inhibitors of α‐glucosidase can effectively suppress the influx of glucose from the intestinal tract to blood vessels resulting in a decrease in postprandial glucose levels (Kim, Nguyen, Kurihara, & Kim, 2010). Therefore, the inhibition of α‐glucosidase can be a good strategy in controlling the blood glucose level in type 2 diabetes. Currently, the traditional antidiabetic drugs such as metformin would effectively attenuate the increase in blood glucose levels, while the long‐term consumption of these drugs is usually associated with multiple undesirable side effects (Chen, Teng, Fang, & Xiao, 2016; Etxeberria, de la Garza, Campion, Martinez, & Milagro, 2012). Antioxidants from natural plant sources could provide an alternative treatment through counteracting the oxidative stress related to an excess of reactive oxygen species (ROS) (Teng, Chen, Fang, Yuan, & Lin, 2017). Such compounds from edible food sources having medicinal properties could offer an attractive strategy to manage diabetes.

*Agaricus blazei* Murill (ABM) is well‐known as a medicinal mushroom. The β‐glucans of ABM are known to stimulate the immune system (Sari, Prange, Lelley, & Hambitzer, 2017; Yamanaka et al., 2012). The antitumor ability of ABM was suggested by Yue et al. (2012) to be related to polysaccharides. In recent years, research has been conducted to obtain high‐purity and high‐activity natural compounds from various types of plants (Coelho et al., 2014; Sheih, Fang, Wu, Chang, & Chen, 2011). The ethanolic extract of buckwheat fermented with ABM showed enhanced antioxidant properties, higher reducing power, better DPPH radical scavenging capacity and ferrous ion chelating ability in comparison to the control without ABM (Kang, Zhai, Li, Cao, & Han, 2017). The polysaccharides from ABM showed antioxidant abilities via scavenging values of DPPH, ABTS and hydroxyl radicals (Jia et al., 2013). ABM ethanolic extract was an effective antioxidative agent which increased the levels of nonenzymatic antioxidants (glutathione, vitamin C, vitamin E) and protected against carbon tetrachloride‐induced oxidative damage in rats (Al‐Dbass, Al‐Daihan, & Bhat, 2012). It also has been reported that ABM demonstrated antidiabetic, antioxidant, blood lipid regulating, and anti‐inflammatory activities (Endo et al., 2010; Souza et al., 2017).

The objective of this study was to identify ABM extracts that may be useful for diabetic care. The antioxidative properties of ABM extracts were evaluated. Additionally, EE and EA were evaluated for potential α‐glucosidase inhibitory activity and improvement of glucose uptake by HepG2 cells.

## 2 MATERIALS AND METHODS

### 2.1 Materials and chemicals

The ABM (dried mushroom) were purchased from Gutian Shenger Food Co., Ltd. 2,2‐Diphenyl‐1‐picrylhydrazyl (DPPH), 2,2′‐azino‐bis (3‐ethylbenzothiazoline‐6‐sulfonic acid) (ABTS), Hydrogen peroxide (H_2_O_2_), trichloroacetic acid, and ferrous sulfate were purchased from Sinopharm Chemical Reagent Co., Ltd. HepG2 cell lines were obtained from Jiangsu KeyGEN BioTECH Corp., Ltd. Minimum Eagle's medium (MEM) and fetal bovine serum were obtained from Hyclone. Trypsin and dexamethasone were obtained from Sigma Chemical Co. Glucose assay kit was purchased from Nanjing Jiancheng Bioengineering Institute. The other laboratory chemicals were of analytical grade.

### 2.2 Plant material and extraction

The ABM (dried mushroom) were crushed (Chinese medicine crusher Model 800Y; Good Kung Fu) and filtered through 80‐mesh screen (SUS304; Olodo, China). The ABM powder was mixed in ratio of 1:30 w/v with pure ethanol and extracted by incubating in a water bath (WNB14; Memmert) at 70°C for 3 hr. After extraction, ethanol extract was filtered through a Buchner funnel and the ABM material was re‐extracted with fresh ethanol for further 3 hr. The solvents were combined and then concentrated to dryness by evaporating with a rotary evaporator (N‐1100; Eyela) and water bath (SB‐1100; Eyela) to remove ethanol. The residue was collected as ethanol extract of ABM (EE). Besides, the ethanol extract was mixed with a threefold volume of ethyl acetate and incubated at room temperature for 2 hr with shaking (MaxQ4450; Thermo Scientific). The ethyl acetate fraction was finally evaporated to dryness at 45°C as the ethyl acetate extract of ABM (EA). The extracts were stored at −20°C until use.

### 2.3 Antioxidant activity assays

#### 2.3.1 DPPH radical scavenging activity

The DPPH radical scavenging activity of the ABM extracts was determined by measuring the absorbance of DPPH (Liao et al., 2012). EE and EA were dissolved in dimethyl sulfoxide at different concentrations (62.5, 125, 250 and 500 µg/ml). A volume of 10 μl of one of the ABM extracts at the above mentioned concentrations was mixed with 190 µl (0.05 mg/ml) of DPPH solution, then vortexed immediately and incubated in the dark at room temperature for 30 min. Absorbance was measured using SpectraMax i3X Microplate reader (Molecular Devices) at 517 nm, using Trolox (Sigma) as a control. DPPH radical scavenging activity was measured using the following equation:

$$\text{DPPH}\;\text{Scavenging activity}\;\left(\%\right)=\left[1-\frac{A_{i}-A_{j}}{A_{0}}\right]\times 100\%$$

where A_0_ is the absorbance of the blank (ethanol without any sample), A_i_ is the absorbance of the sample, and *A_j_* is the absorbance of the control (ethanol without DPPH).

#### 2.3.2 ABTS assay

The ABTS assay was conducted as described previously (Chen & Kang, 2014). Distilled water was used to dissolve ABTS and potassium to final concentrations of 7 and 2.45 mmol/L, respectively. These two solutions were mixed and incubated in the dark at 25°C for 12 hr. The mixture of ABTS and potassium solution was diluted with ethanol to reach an absorbance value of 0.7 ± 0.05 at 734 nm. Next, 190 µl of the mixture was mixed with 10 µl of one of the ABM extracts (EE or EA), and the absorbance at 734 nm was read (SpectraMax i3X Microplate reader) after incubating at room temperature for 6 min, using Trolox (Sigma) as a control. The ABTS radical scavenging activity was measured using the following equation:

$$\text{ABTS}\;\text{Scavenging activity}\left(\%\right)=\left[1-\frac{A_{i}-A_{j}}{A_{0}}\right]\times 100\%$$

where *A*_0_ is the absorbance of the blank (ethanol without any sample), *A_i_* is the absorbance of the sample, and *A_j_* is the absorbance of the control (ethanol without ABTS).

#### 2.3.3 Reducing power assay

The reducing power of EE and EA was quantified as reported previously (Cao, Xia, Dai, Wang, & Xiao, 2014) with some modifications. Briefly, 500 µl of one of the ABM extracts (EE or EA) or Trolox was mixed with 2 ml of phosphate buffer (0.2 mol/L, pH 6.6) and 2 ml of K_3_Fe(CN)_6_ (0.1%, w/v), and incubated at 50°C for 30 min. Next, 1.5 ml of trichloroacetic acid solution (10%, *w*/*v*) was added and immediately vortexed. After centrifugation (2,000 *g*, 10 min), the supernatant (2 ml) was mixed with 2 ml of distilled water and 500 µl of FeCl_3_ (0.1%, *w*/*v*). The absorbance of the solution was recorded at 700 nm using SpectraMax i3X Microplate reader.

#### 2.3.4 Hydroxyl radicals scavenging activity

The hydroxyl radical scavenging activity was measured as described by Liu, Ge, et al. (2017) and Liu, Chen, et al. (2017). Briefly, 4 ml of reaction mixture consisting of 1 ml of the ABM extracts (EE or EA) or Trolox, 1 ml of FeSO_4_ (9 mmol/L), 1 ml of salicylic acid‐ethanol (9 mmol/L), and 1 ml of H_2_O_2_ (9 mmol/L) was incubated at 37°C for 30 min. The absorbance was recorded at 510 nm using SpectraMax i3X Microplate reader. The scavenging capability on hydroxyl radicals was calculated using the following equation:

$$\text{Hydroxyl}\;\text{radical}\;\text{scavenging}\;\text{activity}\left(\%\right)=\left[1-\frac{A_{i}-A_{j}}{A_{0}}\right]\times 100\%$$

where *A*_0_ is the absorbance of the blank (distilled water without any sample), *A_i_* is the absorbance of the sample, and *A_j_* is the absorbance of the control (distilled water without any H_2_O_2_).

### 2.4 α‐Glucosidase inhibition assay

The α‐glucosidase inhibition activity was measured using the method described by Chen and Kang (2014). The α‐glucosidase inhibition activity of EE and EA at different concentrations (10–200 μg/ml) was investigated. A volume of 50 μl of ABM extracts at one of the above mentioned concentrations was added to 50 μl of 5 mmol/L p‐nitrophenyl‐α‐D‐glucopyranoside solution prepared in 100 mmol/L sodium phosphate buffer (pH 6.9) in a 96‐well microplate and incubated at 37°C for 5 min. One hundred microliters of α‐glucosidase (0.1 U/ml in sodium phosphate buffer) was then added to each well and incubated at 37°C for 30 min. Absorbance at 405 nm was recorded and compared to the control which had 100 mmol/L sodium phosphate buffer solution in place of the sample. The inhibition of α‐glucosidase by the ABM extracts was calculated using the following formula:

% Inhibition = 1‐(Absorbance of extract/Absorbance of control) × 100%

### 2.5 Insulin‐resistant HepG2 cell model

HepG2 cells (Jiangsu KeyGEN BioTECH Corp.) were cultured in minimum Eagle's medium (MEM) containing 10% fetal bovine serum (FBS), 1% penicillin and 1% streptomycin in 96‐well plates at 37°C in a humidified atmosphere of 5% CO_2_ for 24 hr. Following this, the medium of normal group was replaced with MEM, while the control group and ABM extracts (EE and EA) treated groups were replaced with MEM mixed with dexamethasone at 2.0 μg/ml for 48 hr. At the end of the treatment, the insulin‐resistant model was obtained. Then, the media of normal group and control group were replaced by MEM, while the ABM extracts treated groups were subsequently replaced with MEM containing different concentrations (10, 50, 100, and 200 μg/ml, respectively) of EE and EA, and incubated at 37°C for another 24 hr.

### 2.6 Cytotoxicity assay

The HepG2 cells were seeded into 96‐well plates at a concentration of 1 × 10^5^ cells/mL and cultured in MEM for 24 hr. When the cells reached confluence, the medium of normal group was replaced by MEM, while the ABM extract‐treated groups were subsequently replaced with MEM containing different concentrations (10, 50, 100, 200, 500, and 1,000 μg/ml, respectively) of EE and EA, and incubated at 37°C for 24 hr. The cell viability was tested using cell counting kit‐8 assay (Beyotime, Shanghai, China) following the manufacturer's instructions. Briefly, 10 μl of CCK‐8 solution was added to each well and incubated for 4 hr at 37°C and the absorbance was then measured at 540 nm using SpectraMax i3X Microplate reader.

### 2.7 Glucose consumption assay

The glucose concentrations in the culture medium were tested using glucose assay kit (Nanjingjiancheng) following the manufacturer's instructions. Glucose consumption was measured using the starting glucose concentration in the culture medium minus the glucose concentration measured at the end of experiment.

### 2.8 Statistical analysis

Results were expressed as means ± standard deviation of data from three repeats. One‐way ANOVA was used for all statistical comparisons among groups, followed by mean comparisons done using the Duncan's multiple‐range test at *p* < .05.

## 3 RESULTS AND DISCUSSION

### 3.1 Antioxidant activity of ABM extracts

**Figure 1:**
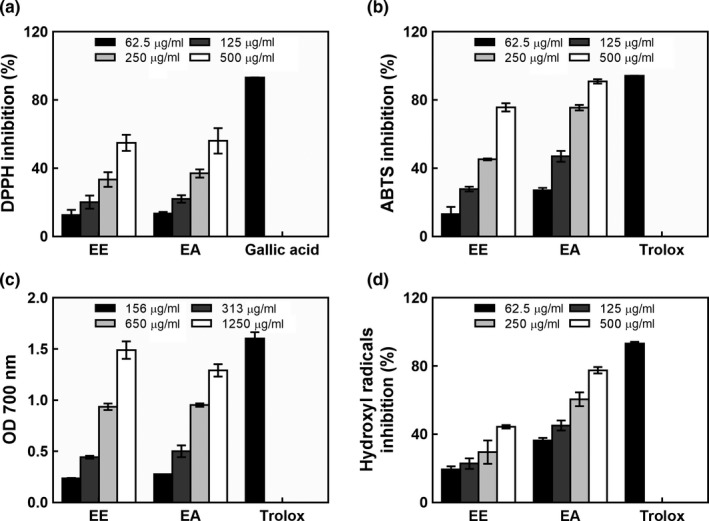
Antioxidant activity of extracts from *Agaricus blazei *Murill: (a) DPPH inhibition; (b) ABTS inhibition; (c) reducing power; and (d) hydroxyl radical inhibition

The high levels of phytochemicals present in edible fungi can contribute to antioxidant activity. The scavenging activity of ABM extracts on DPPH radicals is shown in Figure 1a. The ABM extracts reacted directly with the DPPH radicals and showed increasing scavenging activities at higher concentrations. Overall, the EA showed higher DPPH radical scavenging activity than EE. At 500 μg/ml, the scavenging activity of EE was 54.91 ± 4.71% while that of EA was 56.01 ± 7.49%. There was no significant difference between EE and EA at the same concentration with regard to their DPPH scavenging activity (*p* > .05). The ethyl acetate extract of *Hydnum rufescens* Pers. showed antioxidant activity with an IC_50_ of 852 μg/ml by the DPPH method (Garrab et al., 2019). EA (IC_50_ 421 μg/ml) had better DPPH scavenging activity than the ethyl acetate extract of *Hydnum rufescens* Pers. (IC_50_ 852 μg/ml). Previous studies reported that the antioxidant activity of ABM is attributed mainly to the presence of phenolics and organic content (Carvajal et al., 2012). In the present study, the total phenolic content present in EE and EA was 23 and 36 mg/g, respectively, and these could have potentially contributed to the antioxidant activity.

Using the ABTS assay, the ABTS scavenging ability of ABM extracts was measured at four concentrations, ranging from 62.5 to 500 µg/ml. As shown in Figure 1b, the EA showed an outstanding scavenging activity with an ABTS inhibition value of 90.89 ± 1.27% at 500 µg/ml concentration. The EE was found to have significantly weaker activity (75.69 ± 2.41%) at the same concentration (*p* < .05). Trolox presented a scavenging activity of 94.28 ± 0.04% at a concentration of 62.5 µg/ml. The concentration of EE to inhibit 50% of ABTS was 304 µg/ml. To obtain the same IC_50_ scavenging activity of EA, the concentration of EA was 161 µg/ml. Thus, the IC_50_ value of ABTS for EE was much higher than that exhibited by EA. As the concentrations of EE and EA were increased, the ABTS radical scavenging activity also increased. The EA showed better ABTS radical scavenging activity than EE. The ABTS radical scavenging activity of ethyl acetate extract from *Hydnum rufescens* Pers. with an IC_50_ of 120 μg/ml and the ABTS radical scavenging activity of methanolic extract from *Hydnum rufescens* Pers. with an IC_50_ value of 787 μg/ml were reported by Garrab et al. (2019). In the present study, the ABTS radical scavenging activity of EA (IC_50_ 161 μg/ml) was not as strong as that of the ethyl acetate extract of *Hydnum rufescens* Pers. (IC_50_ 120 μg/ml) (Garrab et al., 2019). However, EE (IC_50_ 304 μg/ml) had better ABTS radical scavenging activity than the methanolic extract of *Hydnum rufescens* Pers. (IC_50_ 787 μg/ml) (Garrab et al., 2019). The reducing ability of ABM extracts is presented in Figure 1c. The results of this study revealed a concentration dependency in the reducing activity of the ABM extracts. The higher concentrations of EE and EA exhibited better reducing activity. EE at 156, 313, 650, and 1,250 µg/ml concentrations exhibited reducing activities of 0.24, 0.44, 0.94, and 1.45, respectively, while EA exhibited reducing activities of 0.28, 0.50, 0.95, and 1.29, at the same concentrations, respectively. A similar reducing ability was found between EE and EA at the same concentrations (*p* < .05). The reducing power of gallic acid was 1.62 at 156 µg/ml. However, the reducing ability of ABM extracts in this study was superior to those demonstrated previously for the reducing ability of ABM ethanolic extract which was 0.37 at 5,000 µg/ml (Tsai, Tsai, & Mau, 2007). These results indicate that extracts from ABM have strong reducing power.

Because of the complex nature of many plants, a single method cannot be used to evaluate the antioxidant activity of extracts from ABM. The scavenging activity of ABM extracts on hydroxyl radicals was analyzed (Figure 1d). Both EE and EA inhibited the hydroxyl radicals efficiently. The higher concentrations of EE and EA showed better hydroxyl radical scavenging activity. The scavenging activity of EE was 44.33% at 500 μg/ml concentration; EA was found to have significantly stronger activity (77.55%) at the same concentration (*p* < .05). The IC_50_ values for EE and EA on scavenging hydroxyl radicals were 602 and 182 μg/ml, respectively. EA showed better hydroxyl radical scavenging activity than EE.

### 3.2 α‐glucosidase inhibition

**Figure 2:**
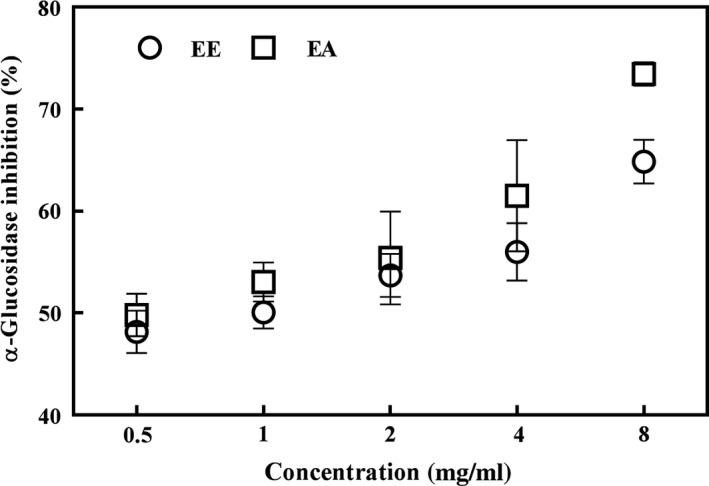
Inhibitory activities of *Agaricus blazei *Murill extracts against α‐glucosidase. EA, ethyl acetate extract; EE, ethanol extract

The α‐glucosidase enzyme can significantly increase the postprandial blood glucose in type 2 diabetes patients. Abnormal function of α‐glucosidase could lead to metabolic diseases. The α‐glucosidase enzyme has been the target of various drugs and inhibitors to regulate the metabolism of sugar in the human body. Therefore, it is important to find natural alternatives to drugs that can inhibit α‐glucosidase (Teng et al., 2017). The α‐glucosidase inhibition capacity of EE and AE in vitro is shown in Figure 2. EE at 0.5, 1, 2, 4, and 8 mg/ml concentrations demonstrated α‐glucosidase inhibition values of 48.15, 50.06, 53.68, 55.99, and 64.86%, respectively, while EA demonstrated α‐glucosidase inhibition values of 49.82, 53.04, 55.39, 61.50, and 73.45%, at the same concentrations, respectively. This shows that both EE and AE had the ability to inhibit α‐glucosidase in a concentration‐dependent manner. At concentrations ranging from 0.5 to 8 mg/ml, EA showed higher inhibitory activity on α‐glucosidase than EE. The results suggested that EA could have high antioxidant capacity and had a relatively higher α‐glucosidase inhibitory activity in comparison with EE. As previously observed, the n‐hexane extract of *Grifola frondosa* showed significant inhibition of α‐glucosidase activity (Su, Lai, & Ng, 2013). The medicinal and edible mushrooms were investigated as α‐glucosidase inhibitors linked to type 2 diabetes. For the α‐glucosidase inhibition, the IC_50_ of methanol extract of *Morchella conica* was 0.521 mg/ml (Stojkovic et al., 2019). The methanol extract of *Morchella conica* showed stronger inhibition of α‐glucosidase than EE (IC_50_ 0.954 mg/ml) used in the present study. These studies show that medicinal and edible mushrooms may have the potential to inhibit α‐glucosidase and thereby contribute to antidiabetic activity.

### 3.3 Cell viability

**Figure 3:**
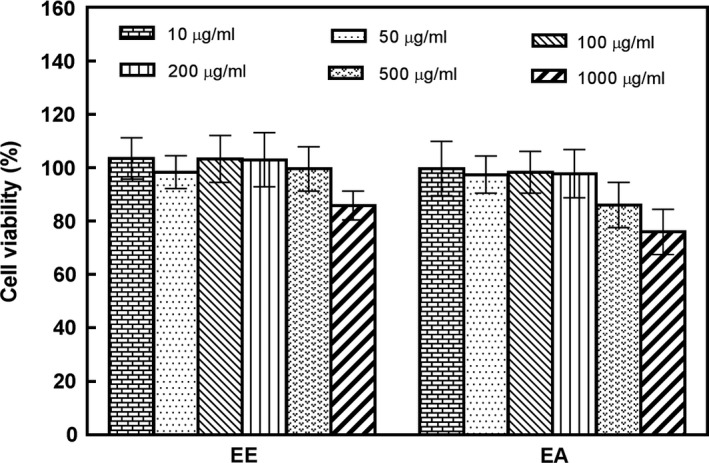
The effect of *Agaricus blazei *Murill extracts on HepG2 cells activity. EE, ethanol extract; EA, ethyl acetate extract

As shown in Figure 3, the potential effects of EE and EA on HepG2 cell viability. EE at concentrations of 10–200 μg/ml demonstrated HepG2 cell survival ranging between 99.2% and 102%, while EA at concentrations of 10–200 μg/ml demonstrated HepG2 cell survival ranging between 96.5% and 99.6%. Above these concentrations, there was a decrease in HepG2 cell survival. Since at concentrations of 10–200 μg/ml, the cell viability assay showed a HepG2 cell survival over 85%, this indicated that treatment with EE and EA at these concentrations did not damage cell integrity during the period of incubation (Piroird et al., 2015). Therefore, 10, 50, 100, and 200 μg/ml extract concentrations were selected for further study.

### 3.4 Glucose consumption by HepG2 cells

**Figure 4:**
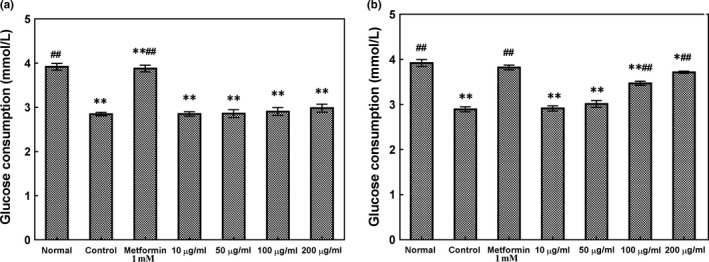
Effects of *Agaricus blazei *Murill extracts on glucose consumption by insulin‐resistant HepG2 cells. HepG2 cells were treated with 200, 100, 50, and 10 µg/ml (a) Ethanol extract or (b) Ethyl acetate extract. Normal represents HepG2 cells without any treatment; control represents HepG2 cells treated with 2.0 µg/ml dexamethasone (a drug that causes insulin resistance in HepG2 cells); Metformin represents HepG2 cells exposed to metformin (a drug that is commonly prescribed to diabetic patients for reducing blood sugar levels) at 1 mmol/L. Compare with normal group, *p < .05, **p < .01; compare with control group, #*p* < .05, ##*p < *.01

The effect of ABM extracts on glucose consumption by HepG2 cells was comparable to that of metformin, one of the first‐line therapeutic agents used to improve hepatic insulin resistance. As shown in Figure 4, the glucose consumption in HepG2 cells was significantly improved by EA treatment. Compared with the control group, EA could significantly increase the glucose consumption by HepG2 cells (*p* < .05) at a concentration of 200 µg/ml and showed no significant differences with metformin at 1 mmol/L (*p* > .05). In contrast to the control group, EE‐treated groups slightly increased the glucose consumption by HepG2 cells at 200 µg/ml, but the difference was not significant compared with the control group (*p* > .05). At the same concentration, EA showed stronger activity on improving glucose consumption by HepG2 cells than EE. The above results suggested that ABM extracts could exhibit an antidiabetic effect. On the whole, hepatic insulin resistance was improved by increasing the glucose consumption by HepG2 cells from extracellular medium. Insulin resistance, also known as impaired insulin sensitivity, results due to the development of tolerance to insulin, making the hormone less effective (Jia, Demarco, & Sowers, 2016; Yan, Gao, Zhang, & Zhang, 2017). Insulin‐resistant glucose uptake is a characteristic feature of diabetes (Ma & Li, 2017; Weickert & Pferffer, 2018). Thus, the regulation of glucose uptake could be beneficial for the prevention of diabetes. An earlier study by Huang et al. (2015) demonstrated that phenolic compounds such as agrimonolide and desmethylagrimonolide showed the inactivation of AMPK pathways, which ameliorated the glucose uptake by HepG2 cells. Oxidative stress refers to the damage caused by excess free radicals and antioxidant defense systems. The severity of type 2 diabetes and its complications are closely related to the state of oxidative stress. Reduction in antioxidant activity coexists with oxidative stress in diabetes which ultimately increases the harmful effects of free radicals. Antioxidants play a significant role in scavenging free radicals, thereby protecting from oxidative stress (Junejo et al., 2018). In this study, ABM extracts showed antioxidant activity by scavenging the free radicals formed in the DPPH, ABTS, and the reducing power assays as well as the hydroxyl free radicals. It is suggested that ABM could potentially be used as antioxidant agents. ABM extracts also had the α‐glucosidase inhibitory property and improvement of glucose consumption by HepG2 cells. Therefore, ABM extracts contributed toward antidiabetic activities both indirectly via their antioxidant properties and directly by inhibiting α‐glucosidase and promoting glucose uptake by HepG2 cells. Thus, it is expected that the intake of ABM may have a beneficial effect in preventing type 2 diabetes.

## 4 CONCLUSIONS

In summary, ABM extracts showed high antioxidant activity in vitro. Results from the DPPH, ABTS, hydroxyl radical scavenging activities, and the reducing power assays showed that EA had better antioxidant activity than EE. The results demonstrated that both EE and EA were capable of inhibiting α‐glucosidase in a concentration‐dependent manner. EA also demonstrated better α‐glucosidase inhibitory activity and improved glucose consumption by HepG2 cells than EE. EA exhibited similar levels of glucose‐lowering activity as metformin. ABM extracts might serve as natural alternatives to drugs such as metformin, since current consumers prefer natural products over synthetic ones. Due to their availability and antioxidant properties, ABM extracts may play a crucial role in functional food applications aimed at glucose‐lowering effect. Although, both ABM extracts showed the potential of glucose‐lowering effect, further studies are needed to understand the mechanisms underlying their antidiabetic activity.

## CONFLICT OF INTEREST

The authors declare that they do not have any conflict of interest.

## ETHICAL STATEMENT

This study does not involve any human or animal testing.
